# Efficiency of Organelle Capture by Microtubules as a Function of Centrosome Nucleation Capacity: General Theory and the Special Case of Polyspermia

**DOI:** 10.1371/journal.pone.0037675

**Published:** 2012-05-25

**Authors:** Ivan V. Maly

**Affiliations:** Department of Computational and Systems Biology, University of Pittsburgh School of Medicine, Pittsburgh, Pennsylvania, United States of America; Stanford University School of Medicine, United States of America

## Abstract

Transport of organelles along microtubules is essential for the cell metabolism and morphogenesis. The presented analysis derives the probability that an organelle of a given size comes in contact with the microtubule aster. The question is asked how this measure of functionality of the microtubule aster is controlled by the centrosome. A quantitative model is developed to address this question. It is shown that for the given set of cellular parameters, such as size and total tubulin content, a centrosome nucleation capacity exists that maximizes the probability of the organelle capture. The developed general model is then applied to the capture of the female pronucleus by microtubules assembled on the sperm centrosome, following physiologically polyspermic fertilization. This application highlights an unintuitive reflection of nonlinearity of the nucleated polymerization of the cellular pool of tubulin. The prediction that the sperm centrosome should lower its nucleation capacity in the face of the competition from the other sperm is a stark illustration of the new optimality principle. Overall, the model calls attention to the capabilities of the centrosomal pathway of regulation of the transport-related functionality of the microtubule cytoskeleton. It establishes a quantitative and conceptual framework that can guide experiment design and interpretation.

## Introduction

Intracellular transport is perhaps the best-characterized function of the microtubule cytoskeleton. Organelles of various types are continually ferried by molecular motors along the cell’s microtubules [1], [2]. The paradigmatic animal cell structure represented by many cell types in culture involves a radial microtubule aster and a dense aggregate of membranous organelles near its center. The radial structure arises from assembly of the microtubules from cytosolic tubulin, which is initiated (nucleated) at the centrosome [3]. The aggregation of the organelles, including prominently the assembly of the morphologically defined Golgi apparatus, arises from the centripetal transport along the microtubules [4]. Transport between the periphery and the center is important, for example, in biosynthetic pathways, where vesicles from the peripheral endoplasmic reticulum are ferried to the centrally located Golgi apparatus [4]. A widely different example is provided by the mechanisms of animal color change, where pigment granules are transported from the periphery to the center, resulting in optical clearing of the cytoplasm [5].

To be transported, an organelle must come in contact with a microtubule. The size of the comparatively large dynein motor complex [6] can be estimated at ∼100 nm, setting the limit for the distance at which the organelle can engage with the microtubule. 100–1000 nm is also the characteristic size, to the order of magnitude, of the transport vesicles and motile organelles. These considerations establish a design constraint on the structure of the microtubule cytoskeleton that will be efficient at its transport function: The spatial density of the microtubules must be sufficiently high in all regions between which the organelles are transported. The density of microtubules near the center of the aster is usually very high. On the order of one hundred microtubules converge there on the pericentriolar material of the centrosome, which has a diameter on the order of a micron [7]. The density of the radial microtubules on the periphery is much lower. Not all microtubules are long enough to reach the periphery, and the spatial density of the longer ones is lowered by their radial divergence [8]. How is this density maintained at levels that are functional? This paper presents a theoretical analysis of how the density of microtubules is regulated by nucleation of their assembly on the centrosome, in the light of the kinetics of microtubule assembly from the cellular pool of tubulin. The goal of this analysis is to establish a quantitative and conceptual framework that makes specific predictions and can guide design and interpretation of new experiments.

It should be noted that additional mechanisms appear to facilitate loading of organelles onto the radial microtubules at the periphery. Non-centrosomal microtubules exist, which are not arranged radially, and which can be present at a higher density at the periphery [8], [9]. At least potentially, they can form a short-distance and non-directional relay system that can supplement the radial system by directing the cargo to the sparser radial microtubules for subsequent long-range transport to the center. An example where this is evidently the case will be considered in detail below (karyogamy in *Beroe*). A similar role of transport along actin filaments has been thoroughly documented in the color-change experimental models [10], [11]. Short-range and nondirectional transport along short actin filaments arranged in an isotropic network is the first stage of pigment granule movement, which is followed by their fast directional transport along the radial microtubules. These short-range mechanisms (in systems where they are active) raise the probability that loading onto the long-range radial tracks eventually takes place. Irrespectively, it can be accepted that the spatial density of the radial microtubules at the periphery is an important parameter that affects the overall efficiency of the transport. It should therefore be controlled by the cellular regulation system.

Regulation of the extent of the microtubule aster has been the subject of quantitative analysis for a long time. Transitions between the asters of long microtubules in the interphase and short ones in mitosis have served as the paradigm for this work. It was determined already in the pioneering model by Mitchison and Kirschner [12] that changes in the nucleation capacity of the centrosome may explain the transition. This explanation is in agreement with the experimental evidence for the inverse correlation between the number and length of the microtubules during the cell cycle and the linked changes in the microtubule dynamics [13]–[17]. Subsequent models of tubulin polymerization in the cell [18]–[22] have elaborated on the kinetic basis of this number-length dependence. The nonlinearity of the kinetics makes it impossible to summarize this effect simply and at the same time fully consistently. Notwithstanding, it can be observed that increased nucleation favors polymerization of tubulin, and the polymerization depletes the cellular tubulin pool. The depletion of the soluble fraction in turn favors depolymerization. Which of these antagonistic effects should prevail, can only be predicted through numerical analysis of the kinetic model. The cited models differ in their detailed assumptions, but they have all predicted that the new steady-state average microtubule length is shorter than it was with the lower nucleation capacity.

Microtubule length can also be regulated by mechanisms that directly affect the kinetic constants of microtubule elongation and shortening [23]–[26]. The regulation through the centrosome [17], [27]–[29] presents a special interest however, because of its indirect nature and the nonintuitive nonlinear behavior of the steady-state tubulin polymerization that mediates it. Generally, the regulation is likely to involve both pathways, further complicating the analysis. Upon fertilization in Metazoa, however, the bulk of the cytoplasm is commonly provided by the egg, and the centrosome for the nucleation of the new aster is provided by the sperm. In fertilized eggs, therefore, the tubulin pool and the conditions that set the elongation and shortening rate constants are established by the female component, and the nucleation capacity for the new microtubules–by the male component. Fertilized eggs may form a useful paradigm for research into the relative roles of the two pathways of microtubule regulation.

Generally, the asters assembled in fertilized eggs guide the movements of the male and female pronuclei that lead to karyogamy [30]. In Ctenophora, this movement involves transport of the female pronucleus along the microtubules radiating from the sperm centrosome to the male pronucleus at the center of the sperm aster [31]–[33]. This case presents special interest, because it can be revealing of how the efficiency of capture and transport by microtubules is controlled by the cell in general. Evidently, the function of the sperm centrosome is to nucleate a microtubule aster that will efficiently capture the female pronucleus. The function of the egg tubulin system, including the tubulin pool and the conditions that set the rate constants of microtubule elongation and shortening, is to ensure that a functional sperm aster is assembled. Thus, there is likely a degree of synergy between the direct and indirect regulation pathways in the fertilized egg. Yet in the case of physiologic polyspermia, such as in the ctenophore *Beroe ovata* [32], the goals of the male and female components become non-identical. The female component, evidently, aims to achieve karyogamy through capture of the female pronucleus by any male microtubule aster. Each male centrosome, however, aims to achieve the capture of the female pronucleus specifically by its own microtubule aster–not by any aster that is present in the polyspermic egg. The control of the microtubule density through nucleation in this case may work not merely independently of, but potentially against the other regulatory mechanisms.

The question that the reviewed line of theoretical work on the regulation of the microtubule length by nucleation has not answered is how the spatial density is affected when the number and length of the microtubules co-vary as prescribed by the polymerization kinetics. This question is addressed in the present paper. It is evident that nucleation has a double-edged effect on the spatial density: more numerous microtubules fill the space more efficiently even as they diverge radially, but their shorter length tempers the increase in the density. Quantitative analysis is necessary to determine which of the antagonistic effects prevails under the given cellular conditions. One of the recent quantitative models of tubulin polymerization [21] is adapted here for this purpose, and some general predictions are derived. The model is then applied to the uniquely revealing case of the physiologic polyspermia.

## Results

### The Steady State

In a previous paper [21], we have demonstrated how the steady-state concentration *c* of unpolymerized tubulin in the cell can be found by solving the following equation (equation 6 in the cited paper):
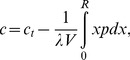
(1)where *c*_t_ is the total concentration of tubulin in the cell, λ = 0.612 nm is the microtubule length increment per 1 tubulin subunit (αβ dimer [34]), and *V* is the cytoplasmic volume. *R* is the limit on the microtubule length (for example, the cell radius, if the radial microtubules abut on the cell margin). *p* is the steady-state number density of microtubules whose length is *x* (equation 5 in the cited paper):
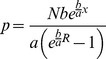
(2)Here, N is the nucleation capacity. In the terminology of the referenced models [12], [18], [21], [35], it equals the number of microtubules, because unoccupied nucleation sites are counted as microtubules of zero length. a is the apparent diffusion coefficient of the dynamic microtubule ends (the second central statistical moment of dynamic instability understood as a stochastic process of microtubule length change). b is the apparent drift coefficient of the dynamic microtubule ends (the first statistical moment of dynamic instability). It is a function of the free tubulin concentration (equation 1 of the cited paper):
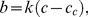
(3)where the rate constant k = 1 µm min–1 µM–1 and the critical concentration cc = 11.5 µM were derived [21] from the experimental measurements of Walker et al. [36]. The value a = 7.5 µm2 min–1 was accepted [21] as representative of the set of direct experimental measurements [9], [37]–[39].

The equation for the concentration was solved numerically in the cited prior work. Here it is observed that when the microtubule growth is not restricted geometrically, the equation admits an analytical solution. This case is of interest, because in various cell types, microtubules grow along the cell boundary instead of abutting on it. The egg of *Beroe*, which is modeled in subsequent sections, is one example. In the limit of *R*→∞ (microtubule length unrestricted by the cell structure), Equation 2 becomes
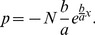
(4)Evidently, for Equation 4 to be physical, *b* must be negative. This stipulates that the steady-state concentration is lower than the critical one, according to Equation 3. That it must be so in the absence of a specially imposed limit on the length was observed already by Oosawa [40] and Hill [41]. Substituting Equations 3 and 4 into Equation 1, we obtain
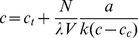
(5)The solution is



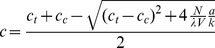
(6)It can be observed from Equation 6 that the normalized deviation of the steady-state concentration from critical obeys an especially simple law:
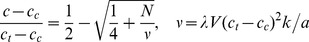
(7)This form of the solution (Equation 7) also makes it obvious why the other root of Equation 5, which would have a plus sign before the square-root sign, is nonphysical. Indeed, assuming that the total concentration is above critical, the steady-state concentration would then be above critical, and Equation 4, invalid.

Equation 7 reveals the nonlinearity of the dependence of the steady-state concentration of the unpolymerized tubulin on the nucleation capacity. In the limit of zero nucleation capacity, the unpolymerized concentration reaches critical. The rate of the concentration change with *N* is controlled by ν (Equation 7). This dimensionless compound parameter can be regarded as the natural unit of *N*, from the standpoint of how *N* regulates the state of tubulin polymerization in the cell: Small deviations of the concentration from critical are predicted when *N* is small compared with ν.

**Figure 1:**
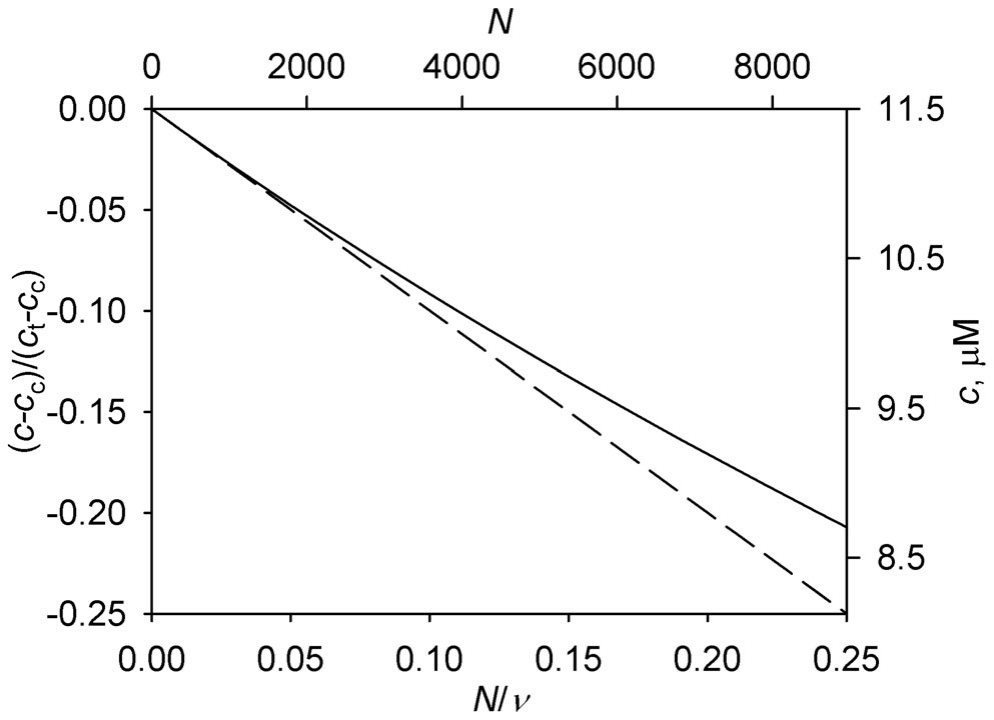
Dependence of the steady-state concentration of unpolymerized tubulin *c* on the nucleation capacity *N*. Solid curve, fundamental solution according to Equation 7. Dashed curve, approximation according to Equation 9. One set of axes presents the functions in their parameter-invariant form. The other presents them in units that correspond to the parameter values derived previously for a “generic” animal cell [21]. See Table 1 for parameter definitions and values. For illustrative purposes, the abscissa range is extended beyond values that should be realistic for the generic cell.

The derivative of the normalized concentration (Equation 7) with respect to *N* is
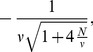
(8)which equals –1/ν, when *N* = 0. Therefore, an especially simple expression for the normalized concentration is valid when *N* is small compared with ν:
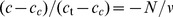
(9)
Figure 1 compares the calculation according to the fundamental expression (Equation 7) and its approximation (Equation 9). It can be seen that the approximation remains accurate throughout the range of N that may be considered realistic for a generic animal cell.

**Table 1:** Parameters.

Symbol	Parameter	Value
λ	microtubule length per tubulin subunit	0.612 nm [34]
*a*	diffusion coefficient of microtubule ends	7.5 µm^2^/min [9], [37]–[39] *
*c*_t_	total concentration of tubulin	25 µM [42], [43] *
*c*_c_	critical concentration of tubulin	11.5 µM [36] *
*d*_e_	diameter of the *Beroe* egg	1 mm [32]
*d*_f_	diameter of the female pronucleus	15 µm [33]
*h*	thickness of egg ectoplasm	5 µm [33]
*k*	microtubule elongation rate constant	1 µm min^–1^ µM^–1^ [36] *
*N*	number of microtubules nucleated at the centrosome	varying
*N*_f_	number of acentrosomal microtubules in the egg	varying
*N*_m_	number of microtubules nucleated at the sperm centrosome	varying
*n*	sperm number per egg	varying
*V*	cell volume	4 pL [18], [43] * †

* Reference [21] derives this estimate from the experimental data in the papers cited here. See text.

† This typical value does not apply to the large *Beroe* eggs, whose ectoplasmic volume is calculated from *d*_e_ and *h*. See text.

### Microtubule Density

**Figure 2:**
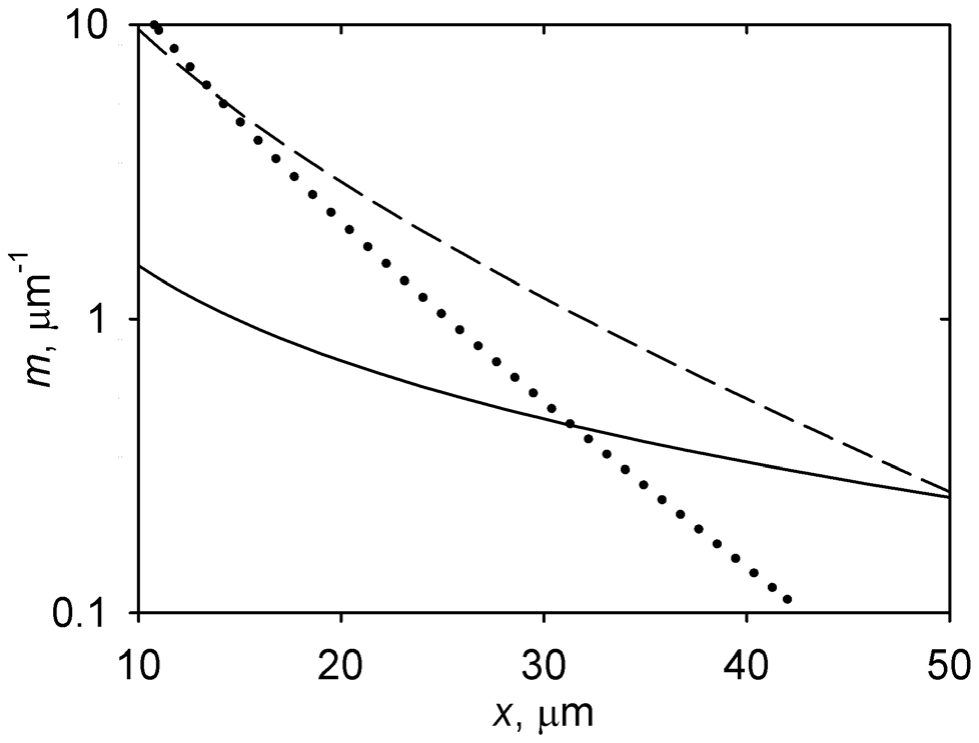
Regulation of the microtubule density profile *m*(*x*) by the centrosome nucleation capacity *N*. Solid curve, *N* = 100; dashed curve, *N* = 1000; dotted curve, *N* = 2000. Calculated according to Equation 10 and accepting the parameter values from Table 1.

The spatial density of microtubules at the distance *x* from the center of the flat radial array can be defined as the number of microtubules that cross the circumference of radius *x*, per unit arc length. The number of microtubules crossing such a circumference is the number of microtubules whose length is greater than *x*. The steady-state microtubule density can therefore be obtained as follows:
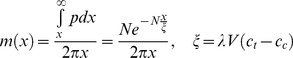
(10)Here, Equation 4 was used as the expression for *p*, and Equations 3 and 9 were substituted to express the steady-state *p* as a function of *N*. Equation 10 reveals that the only kinetic parameter that controls the microtubule density is the critical concentration (*c*_c_). Increasing the nucleation capacity raises the microtubule density primarily closer to the centrosome, and reduces the microtubule density primarily in the more distant regions (Figure 2).

**Figure 3:**
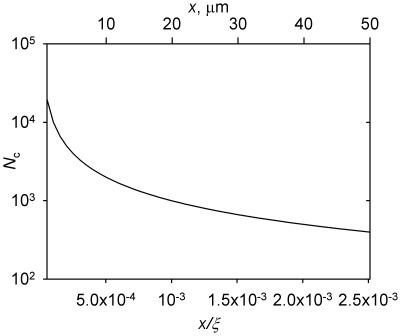
Nucleation capacity *N*_c_ at which the microtubule density at the distance *x* attains the maximum. Calculated according to Equation 12. The bottom axis presents the function in its parameter-invariant form. The top axis is in units that correspond to the parameter values derived previously [21] and accepted here for a “generic” animal cell (Table 1).

The derivative of the microtubule density (Equation 10) with respect to *N* is
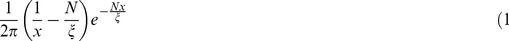
(11)Its sign is determined by the expression in brackets. It is positive at zero *N*, reflecting the intuitive expectation that the microtubule density grows with the nucleation capacity of the centrosome. However, the second term in it grows with *N*. This means that the microtubule density will decline with *N* for sufficiently large *N*. The transition from the growth to decline takes place when *N* is equal to the root of the expression in brackets in Equation 11,



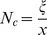
(12)The optimal value of *N* (*N*_c_, Equation 12) is inversely proportional to the distance from the centrosome (Figure 3). Thus, the density of microtubules in different parts of the cell can be differentially regulated by the nucleation capacity of the centrosome. It is possible to maximize the density at a certain position by setting the nucleation capacity according to Equation 12, but this will be achieved at the expense of the microtubule density elsewhere.

### Organelle Capture

The capacity of the microtubule system to initiate the transport of an organelle is determined by the probability that the organelle is in contact with at least one microtubule. In the case of the effectively two-dimensional array of microtubules, which was considered in the last section, the probability of contact (“capture”) can be approximated as the probability that at least one microtubule crosses the circle that represents the dimensions and the position of the organelle. The diameter of the circle, *d*_o_, can be assumed to be small compared with the dimensions of the cell and with the mean microtubule length, as well as with the distance from the centrosome. Under these conditions, the probability in question is approximated well by the probability *P*(x) that at least one microtubule crosses an arc length *d*_o_ of the circumference whose radius *x* equals the distance between the centrosome and the organelle. If the directions of the microtubules are random, then the number of microtubules crossing such an arc will have the Poisson distribution. Its mean will be *m*(*x*)*d*_o_, where *m*(*x*) is the microtubule density from Equation 10. The probability that the number of the crossing microtubules is not zero will be
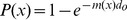
(13)


**Figure 4:**
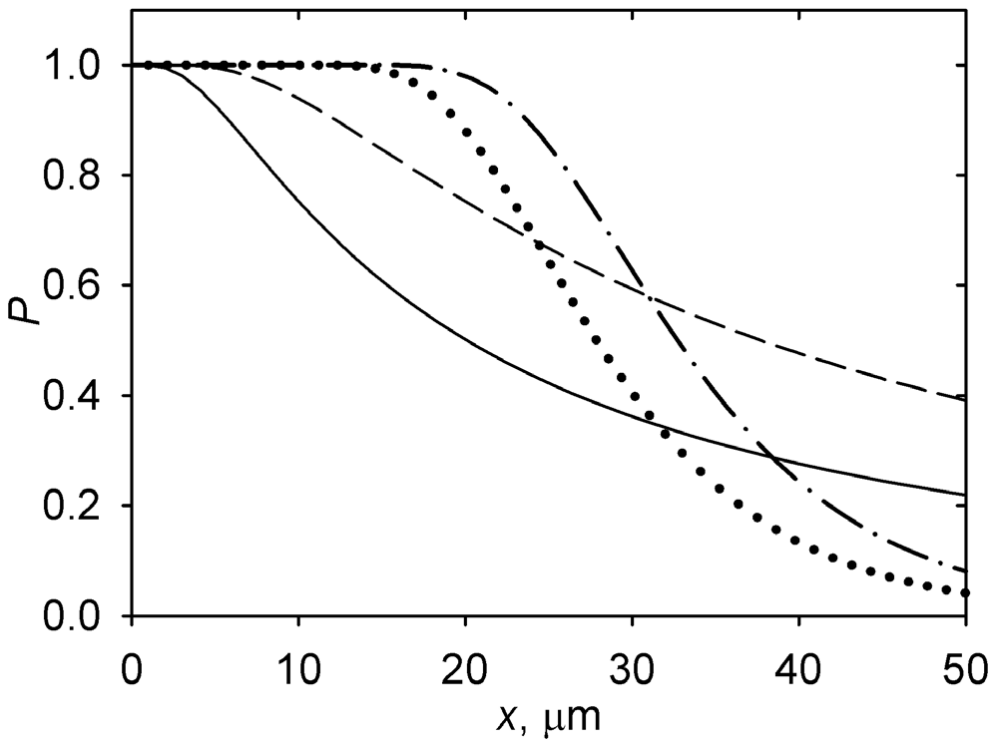
Probability of organelle capture *P* as a function of distance from the centrosome *x*. Calculated according to Equation 13 and accepting the parameter values from Table 1. Solid curve: *N* = 100, *d*_o_ = 1 µm. Dashed curve: *N* = 100, *d*_o_ = 2 µm. Dotted curve: *N* = 2000, *d*_o_ = 1 µm. Dash-dot curve: *N* = 2000, *d*_o_ = 2 µm.

The shape of this function and its control by *N* and *d*_o_ are illustrated in Figure 4. Comparison of Figures 2 and 4 demonstrates that *P* approaches 1 (certainty of capture) near the centrosome, where the microtubule density is very high. The dependence of *P* on *d*_o_ is direct and qualitatively predictable (Figure 4).

Figure 4 further demonstrates that increasing *N* raises the probability of capture in regions closer to the centrosome and reduces it in more distant regions. This is similar to how *N* controls the microtubule density (Figure 2). Indeed, since *P* (Equation 13) is a monotonically increasing function of *m*, the analysis presented in the last section applies also to *P*: Specifically, the probability of capture will increase with *N* for *N*<*N*_c_, and decrease for *N*>N_c_, where *N*_c_ is defined as a function of *x* by Equation 12. Thus, the structure of the radial microtubule array may be optimized for capturing organelles at a given distance *x* by setting the nucleation capacity of the centrosome.

In addition to the qualitative similarity between the regulation of *m* and *P*, there is an important quantitative difference. Due to the discussed saturation of *P*, regulation of *m* in regions near the centrosome becomes comparatively inconsequential. This effect reduces the practical significance of the dependence of the optimal nucleation capacity on the distance from the centrosome, because this function (Equation 12) changes most rapidly at the short distances (Figure 3). Thus, as compared with the microtubule density, the probability of organelle capture can be maximized everywhere relatively uniformly by setting the appropriate value of *N*.

### The Egg of *Beroe*

The egg of *Beroe ovata* is approximately spherical and has a diameter of approximately *d*_e_ = 1 mm [32]. Its volume is mostly occupied by yolk. The thickness *h* of the outer layer of cytoplasm (ectoplasm), which is occupied by microtubules and organelles, is from 5 to 30 µm [33]. It can be tentatively accepted that the lower bound of this range is representative of the average thickness (*h* = 5 µm), while the higher values are observed near the comparatively large pronuclei and do not significantly affect the volume of the ectoplasm. Given the size of the egg, the ectoplasm may be considered effectively two-dimensional as far as the spatial distributions are concerned. Its physical volume can be calculated as π*d*_e_^2^ *h* = 15.7 nL. This volume is very large compared with the volumes of typical cells in culture, which are ∼1 pL [21]. It can be tentatively accepted that the unpolymerized tubulin also is present only in this volume, and not in the yolk. It can also be accepted as a hypothesis that the total tubulin concentration *c*_t_ in the ectoplasm is the same as the average [21] between the experimental measurements made in a variety of cells, 25 µM [42], [43].

A large number of acentrosomal microtubules are present, which are arranged isotropically under the egg surface within the ectoplasmic layer [33], [44]. Since these belong entirely to the female part of the cytoskeleton in the fertilized egg, their number will be denoted *N*_f_. Upon fertilization, microtubule asters assemble around the sperm centrosomes next to the male pronuclei. The number of microtubules per aster will be denoted *N*_m_, which in the terminology accepted here [12], [18], [21], [35] is also the nucleation capacity of the sperm centrosome. The eggs develop normally when the number *n* of sperm per egg is 1–10, which is a normal and physiologic level of polyspermia in *Beroe ovata* [32], [45]. The female pronucleus has a diameter *d*_f_ = 15 µm. It is captured by the sperm asters and is transported along their microtubules centripetally to fuse with the male pronucleus near the centrosome [33]. The summary of these parameter values and those that have been accepted in the preceding sections can be found in Table 1.

*N*_f_ and *N*_m_ remain free parameters. About their values it can only be said on the basis of the published data [33], [44] that they probably lie within the 10^2^–10^6^ range. The lower bound represents values typical of generic and well-studied cells in culture [21], and the upper bound is chosen to be very large but still plausible. The absolute magnitude of these values is large, given that the egg of *Beroe* is very large. However, it should be observed that with the parameter values accepted here for the *Beroe* egg, using Equation 7 one computes ν = 1.4⋅10^8^. This exceeds by two orders of magnitude the estimated upper bound on the total nucleation capacity in the egg. Therefore, in terms of its impact on the state of tubulin polymerization, the reasonable upper limit on the nucleation capacity in the egg is still “small”, and the simple expression for the steady-state tubulin concentration (Equation 9) applies.

The simple radial divergence in two dimensions, which was considered in the preceding sections, is not exactly applicable to the spherical ectoplasm. It would be an acceptable local approximation of the form of the male asters at distances *x* from their respective centrosomes that are small compared with *d*_e_. However, the microtubules in the male asters can extend to several hundred micrometers [33], which is a significant fraction of the egg size. It is therefore necessary to capture the global geometry of the ectoplasm layer in the model. This is achieved by replacing the denominator 2π*x* in Equation 10 with π*d*_e_sin(2*x*/*d*_e_). This expression gives the length of the circumference whose geodesic distance from the centrosome (as the microtubules run under the egg surface) is *x*. Additional changes to the generic form of the microtubule density function (Equation 10) are needed to incorporate the breakdown of the nucleation capacity *N* into the egg-specific parameters *N*_f_ and *N*_m_. Indeed, all microtubules are at steady state with the same pool of tubulin, but only the ones diverging from the sperm centrosome contribute to the microtubule density in question. Applying all the above considerations to modify Equation 10, we define the microtubule density function *m*_e_, which is specific to the *Beroe* egg:
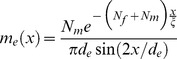
(14)


The problem of the capture of the female pronucleus in *Beroe* also has some specific characteristics compared with the general problem of organelle capture considered in the last section. There is only one female pronucleus in the egg, and its location with respect to the sperm centrosome is random due to the random sperm entry [31], [32], [44]. Given the random distribution on the spherical surface of the egg, the probability density that the distance between the male centrosome and the female pronucleus is *x* will be sin(2*x*/*d*_e_)/*d*_e_. The probability density that the female pronucleus is captured at the distance *x* from the sperm centrosome is given by the product of the probability *P*(*x*) (Equation 13) and the probability density that the distance between the male centrosome and the female pronucleus is *x*. The total probability of capture will be obtained by integrating the said product over all possible *x*:
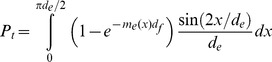
(15)It can be seen that Equation 15 would not be accurate if microtubules longer than one-half of the egg’s circumference would make a significant contribution to the microtubule density. Such microtubules are unlikely to exist in *Beroe* eggs, according to the data presented in the cited papers.

**Figure 5:**
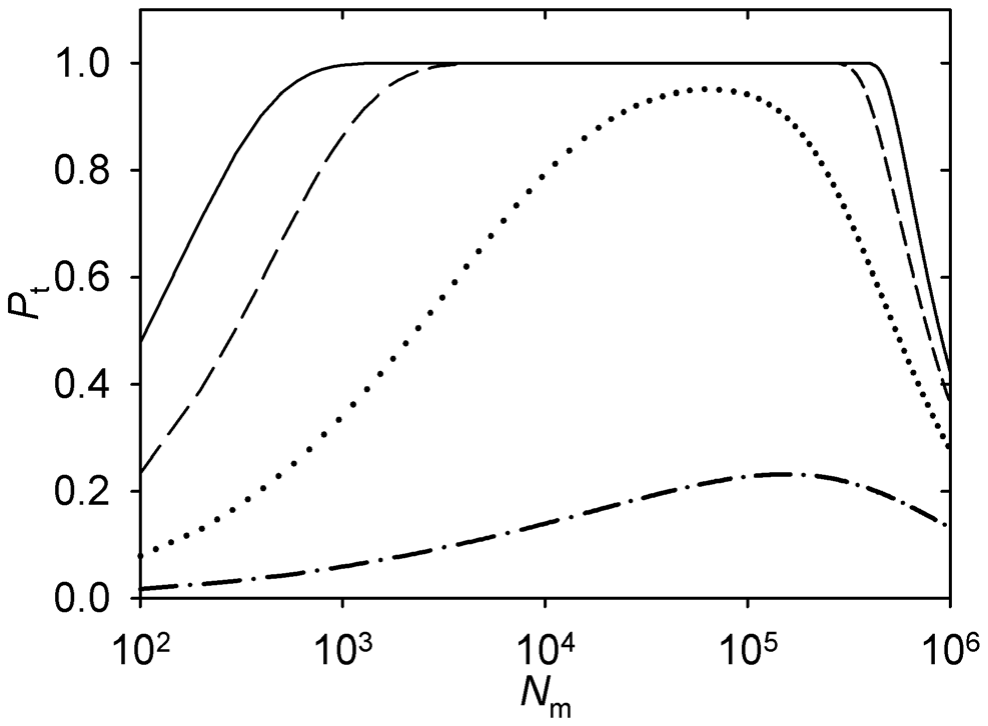
Probability of pronucleus capture *P*_t_ as a function of the sperm centrosome nucleation capacity *N*_m_. Calculated according to Equation 15 with parameter values accepted for the *Beroe* egg (Table 1). Solid curve, *N*_f_ = 10^2^. Dashed curve, *N*_f_ = 10^5^. Dotted curve, *N*_f_ = 3⋅10^5^. Dash-dot curve, *N*_f_ = 10^6^.

*P*_t_ as a function of *N*_m_ can be computed numerically for different values of *N*_f_ (Figure 5). The calculations reveal that in most of the range of conceivable values of *N*_f_ (10^2^–1⋅10^5^), a broad range of *N*_m_ (10^3^–10^5^) results in *P*_t_ near 1, i.e. a guaranteed successful capture of the pronucleus. Above *N*_f_ = 1⋅10^5^, the probability as a function of *N*_m_ begins to exhibit a uniquely defined maximum. The maximum at first remains near 1, but as *N*_f_ reaches 10^6^, it drops to levels that are so low (Figure 5) that the capture should become dependent primarily on the random rather than the directional movement of the pronucleus [33]. Overall, the model predicts that the kinetics of tubulin polymerization in the egg constrains the nucleating capacity of the centrosome with which the optimal extent and density of the sperm microtubule aster can be achieved. Depending on the number of pre-existing acentrosomal microtubules in the egg, the optimal nucleation capacity of the sperm centrosome can lie within a significant range of values or be defined uniquely.

### Polyspermia

According to the analysis put forward in the Introduction, physiologically polyspermic eggs may be uniquely revealing of the mechanisms of regulation of the microtubule cytoskeleton by the centrosome. It is reasoned that in polyspermic eggs the goal of the regulation by the individual sperm centrosome may differ from the function served by the rest of the tubulin polymerization system, which is formed by the female components of the egg. What should matter from the perspective of the given sperm is the probability that its own aster captures the female pronucleus, irrespective of the total probability that it is captured by any of the asters that are present.

When there are *n* asters in the egg, the density of microtubules of the given aster (*m*_a_) is defined by the following variation of Equation 14:
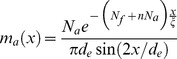
(16)Here, *N*_a_ is the number of microtubules in each of the *n* asters present in the egg. Equation 16 reflects the fact that microtubules of all *n* asters, as well as the noncentrosomal microtubules, are included in the nucleation capacity insofar as it defines the steady-state tubulin concentration. Only the microtubules radiating from the given centrosome are included, however, in the spatial density of the respective aster. Substituting *m*_a_ (Equation 16) for *m*_e_ in Equation 15 yields the probability *P*_a_ that the pronucleus is captured by the given aster, out of *n*.

**Figure 6:**
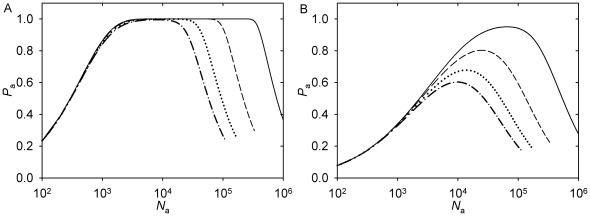
Probability of the female pronucleus capture by a given sperm aster in a polyspermic egg. See Table 1 for the parameter values accepted for the *Beroe* egg. Solid curve, *n* = 1. Dashed curve, *n* = 3. Dotted curve, *n* = 6. Dash-dot curve, *n* = 9. (A) *N*_f_  = 10^5^. (B) *N*_f_  = 3⋅10^5^.

Results of the numerical computation of *P*_a_ as a function of *N*_a_ are plotted in Figure 6. They have a different form, depending on whether *N*_f_ lies in the range where probability 1 is reached. When probability 1 is reached (with lower *N*_f_), the effect of the sperm number is to contract the range of *N*_a_ in which the maximum probability is reached (Figure 6A). Notably, the range is contracted from the right: Higher nucleation capacities per sperm centrosome, which may be optimal with lower sperm numbers, are no longer optimal with higher sperm numbers. When probability 1 is not reached (with higher *N*_f_), the effect of the sperm number is reduction of the uniquely defined *N*_a_ which is optimal from the perspective of the individual sperm (Figure 6B). The predictions that the maximum probability of capture by the given aster is reduced with *n*, and that the range of *N*_a_ at which the certainty of capture is reached shrinks with *n*, are trivial in the conceptual framework provided by the model. In contrast, the prediction that the optimal *N*_a_ is reduced, or that higher values of *N*_a_ that were optimal are no longer optimal at higher sperm numbers is non-trivial. Whether it is deemed counterintuitive or not, it serves to demonstrate the existence of the optimal nucleation capacity in a way that cannot be conflated with the possible functions of the rest of the microtubule regulation system.

## Discussion

The presented analysis predicts how the density of microtubules is regulated by nucleation of their assembly on the centrosome. According to the model, the kinetics of microtubule assembly from the cellular pool of tubulin constitutes a constraint that links the nucleation capacity and the efficiency of organelle capture by the microtubule system. This work establishes a quantitative conceptual framework that calls attention to the role of the centrosome in regulating the efficiency of the intracellular transport. The predicted quantitative relationship can be tested by modulating the centrosome nucleation capacity and measuring the time before initiation of long-range centripetal transport of organelles on the periphery of the cell. Methods for directly monitoring the movement and detecting the transition to the long-range transport on radial microtubules are well-developed [10], [33], [46]. The nucleation capacity can conceivably be modulated by a tempered application of methods that have been used for its complete suppression (e.g., [46]–[48]). It is hoped that experimental tests of the new quantitative predictions, and, in general, experimental work in the quantitative framework set by the model, will shed additional light on the centrosomal pathway of the cytoskeleton regulation.

The predictions concerning how the spatial density is affected when the number and length of the microtubules co-vary as prescribed by the polymerization kinetics are derived here for the first time. In this respect the new work extends the line of inquiry into the regulation of the cellular tubulin partitioning between monomer and polymer by the nucleation capacity [12], [18], [19], [21], [22], [40], [41]. The key new prediction is that a level of nucleation capacity exists that maximizes the microtubule density at the given distance from the centrosome (Equation 12). It is also demonstrated that an optimal nucleation capacity exists that maximizes the probability of capture of an organelle. The optimal nucleation capacity can be calculated for capturing an organelle positioned at a given distance from the centrosome (Equation 13), or positioned randomly in the cell (Equation 15).

As was pointed out in the Introduction, the case of physiologic polyspermia is especially revealing of the role of the centrosomal pathway of regulation. Indeed, the centrosome nucleation capacity is but one parameter that may affect the cytoskeleton structure and functionality. Yet in polyspermic eggs, regulation via this pathway is conceivably pursuing a goal that is not identical with the goal of the rest of the tubulin regulation system. The prediction that might appear counterintuitive outside the new model framework is that the sperm centrosome in physiologically polyspermic species should have a lower nucleation capacity. It might be expected from qualitative considerations that a lower nucleation capacity, per sperm, would be sufficient to achieve karyogamy in these cases. The new theory, however, predicts that a lower nucleation capacity is also necessary for the given sperm centrosome to maximize the probability that the karyogamy involves its respective male pronucleus. Thus, apart from being an example of applying the new modeling apparatus to a specific cell type, the model for the egg of *Beroe ovata* that is presented here sharpens the argument that the functionality of the microtubule array can be optimized through regulating the centrosome nucleation capacity.

The model reveals that the numerous cellular and kinetics parameters describing the tubulin polymerization system do not control the steady-state functional structure of the microtubule aster individually. Instead, as the model showed, the steady-state tubulin concentration and microtubule density are controlled by two specific combinations of the basic parameters, ν and ξ (Equations 7 and 10). ν is non-dimensional and can be regarded as the natural unit of the nucleation capacity, as far as the regulation of the steady-state concentration is concerned (Equation 7). ξ has units of length and controls the microtubule density (Equation 10). It can be regarded as a natural unit of length as regards the cellular tubulin system: ξ would be the total length of the cell’s microtubules, if the unpolymerized tubulin concentration would be equal to the critical one. It should be pointed out that there is a simple and natural relationship between the two: νχ = ξ, where χ = *a*/(*k*(*c*_t_–*c*_c_)). According to Eq. 4, χ would be the average microtubule length in the aster, if the free tubulin concentration would be equal to the difference of the total and critical one.

Thus, the apparent complexity of the purely descriptive traditional multiparametric description of the cellular tubulin system can be reduced to the considerably fewer naturally defined constants. Three kinds of predictions stem from this reduction of complexity in the light of the model. Firstly, the effects of experimental or regulatory changes of the individual cellular or kinetic parameters will be equivalent, if they lead to the same changes in ν and ξ. The equivalency can be checked by mere arithmetic according to the definitions of these constants (Equations 7 and 10). Secondly, experimental or regulatory changes in the multiple individual parameters that are coordinated in a way that leaves ν and ξ unaffected because of their mutual compensation, will have no impact on the steady-state system and its functionality. How they can be so coordinated is, again, prescribed by the definitions of ν and ξ. Finally, apart from the regulation and experimentation, the corollary of these predictions is that the individual parameters are free to change in the course of the evolution, insofar as their evolution is linked as prescribed by the constancy of ν and ξ.

The model for the *Beroe* egg that is constructed here deals with a specific functional aspect of the cytoskeleton structure: the effective coverage of the ectoplasmic space by the sperm asters and their corresponding efficiency at capturing the female pronucleus. The model accounts for the presence of the acentrosomal microtubules inherited from the oocyte and for the possible multiplicity of the sperm asters. Although it thus addresses some select aspects of the structural basis for the pronucleus migration and karyogamy, the model presented is not intended to be a model for these motility phenomena as such. It does not make any prediction regarding a number of interesting aspects of the intracellular dynamics that follow fertilization in *Beroe*. For example, failure to fuse and subsequent centrifugal movement and apparent transfer to another male aster are observed in some cases [32], [33]. It is also apparent that the female microtubules play a role in the constitutive random transport of the female pronucleus [33] that is similar to the role of actin filaments in some other cell types, as reviewed in the Introduction. The present model for the cytoskeleton structure does not address the physical basis of the trajectory of the female pronucleus. It attempts to predict only how the efficiency of capture by the sperm asters is regulated by the nucleation capacities of their centrosomes. An interesting possibility for which the present steady-state model cannot account is that the movement of the pronucleus is initiated before the steady state of tubulin polymerization in reached in the polyspermic egg. If this is the case, the sperm that entered earlier may hold an advantage, because the subsequently entering sperm will have to initiate nucleation in a lower free tubulin concentration.

The assumption of confinement of the tubulin system to the essentially two-dimensional ectoplasm of the *Beroe* egg warrants additional discussion. Confinement of the microtubules is experimentally documented [33]. The thickness of the ectoplasm, as measured in the same study, is incomparably smaller (microns) than both the size of the egg (1 mm, Table 1) and the characteristic extent of the astral microtubules, hundreds of microns [33]. As far as the spatial distribution of microtubules, therefore, they are effectively confined to a two-dimensional spherical surface. In this regard, the assumption is merely an accurate geometrical approximation. In regard to the estimation of the effective volume, which enters the calculations for the tubulin concentration change, the decision to use not the entire volume of the sphere but only the volume of the thin spherical shell (the ectoplasm) is a strong assumption. The reasoning is that if the non-polymerized tubulin and the microtubules are both confined to the thin ectoplasm, then the tubulin system of the *Beroe* egg is similar to the thinly spread cultured cells on which most of the microtubule research has been done. Although the ectoplasm is many times larger, the ratio of its linear dimensions to the volume is similar to the typical cultured cell. If, however, the microtubules are confined to the spherical shell but the unpolymerized tubulin is distributed in the entire volume of the egg, then the system is qualitatively different. At the same concentration (which affects the assembly kinetics) there will be an extremely large amount of tubulin, relative to the linear dimensions within which the microtubules can grow. This was deeemed unlikely, and the volume calculation was based the on the ectoplasm alone.

The power of the analytical framework of the model can be further demonstrated by considering the variability of the total tubulin concentration *c*_t_ and the uncertainty of the applicability of the previously derived average estimate to the egg of *Beroe. c*_t_ enters the calculations for the *Beroe* egg through the parameter ξ in Equation 14 (and its variant for polyspermia, Equation 16). According to its definition in Equation 10, ξ is proportional to the difference of the total and critical concentrations, *c*_t_–*c*_c_. If *c*_t_ varies between 20 and 30 µM (see references from Table 1), then *c*_t_–*c*_c_ varies between 0.63 and 1.37 of the value assumed based on *c*_t_ *c*_t_ = 25 µM. ξ enters Equations 14 and 16 in the denominator of the exponent, and the total number of microtubules enters the numerator. Accordingly, varying the pre-existing number of microtubules *N*_f_ by a factor of 0.63 to 1.37 would exactly compensate for the possible variation in *c*_t_, and exactly the same calculation results would be achieved as far as the microtubule density and the probability of capture. This is a very small range of variation compared with the uncertainty in *N*_f_: the analyzed range of *N*_f_ in Figure 6 is three-fold and in Figure 5 it is ten-thousand-fold, amply covering the effective uncertainty of ξ that is arising from the uncertainty in *c*_t_ (merely 1.37/0.63 = 2.18-fold). These considerations illustrate how the new analytical model can be used to reason about the effects of cell parameter changes without actually evaluating the model numerically.

The largely analytical model derived here relies on the analytical solution (Equation 5) of Equation 1 that is valid only when the microtubule growth is not limited by the dimensions of the cell. This applies to the eggs of *Beroe*, for example, but is not valid in general. To extend the model to the case where growing microtubules abut the cell boundary instead of growing along it, the formalism derived here must be applied to a numerical solution of Equation 1. Previously we demonstrated how this equation can be solved numerically in the general case [21]. Once the steady-state *c* is known, it can be plugged into the general expression (Equation 2) for the microtubule length density function *p*, instead of the special-case expression (Equation 4). This *p* can then be used to derive the microtubule density as prescribed by Equation 10, and the probability of organelle capture as prescribed by Equation 13.

It is not inconceivable that the optimal nucleation capacity, whose theoretical existence is revealed by the model, may be reached through dynamic regulation of the centrosome in the given cell that is adapting to its function. For example, it is not entirely inconceivable that the sperm centrosomes somehow sense the egg’s sperm number and adapt to it dynamically. The alternative that may be simpler is that the nucleation capacity of the given cell type is controlled genetically and is optimized by the evolution of the species. A physiologically polyspermic species of Ctenophora, such as *Beroe ovata*, would then constitutively have a lower nucleation capacity of the sperm centrosome compared with a species in which polyspermia is not normal.

The analysis presented in this paper demonstrates further the utility of the diffusion-with-drift approximation of the stochastic process of microtubule assembly. The stochastic nature of tubulin polymerization makes the process of microtubule assembly complex and incompletely characterized on the molecular level. Two frameworks have been used to describe quantitatively its phenomenological appearance on the light-microscopic level: the dynamic instability model with 4 or 8 parameters [15], [49]–[52], and the diffusion-with-drift model with 2 parameters [9], [21], [35], [37]–[39], [41], [52]–[54]. Apart from being the framework for the experimental measurements in the cited papers, both models have been used (somewhat interchangeably) to make quantitative predictions concerning the cytoskeleton structure and regulation (e.g., [12], [18], [19], [21], [22], [37], [41], [53]–[54]). The theoretical connection between the two has been expounded in the previous literature [21], [41], [52]–[54]. The simpler diffusion-with-drift model is an approximation of the dynamic instability model that is generally valid on the cell scale [52], and, as the research practice shows, both models are reasonably accurate approximations of the actual complex process of stochastic reversible microtubule assembly.

The basis for the main predictions in this paper is the quantitative law of partitioning between monomer (i.e., αβ dimer) and polymer in the cellular tubulin system. Previously, the tubulin partitioning has been characterized by means of Monte Carlo simulations [18], [19], [22] and other numerical algorithms [12]. These methods were required to make predictions using the dynamic instability approximation. In the general case, numerical solution of the equations is also indispensable when the diffusion-with-drift model is used [21]. In the present paper, it is demonstrated that a simple and revealing analytical solution exists, when the diffusion-with-drift description is applied to the mathematically special but biologically common case, wherein microtubules can grow along the cell boundary instead of abutting on it. The analytical solution for the steady-state concentration of unpolymerized tubulin as a function of the centrosome nucleation capacity makes the subsequent analysis of density and capture more revealing and informative compared with numerical examples that could exclusively be obtained otherwise.

Like previous models of this type (e.g., [12], [21], [37], [41], [52]–[54]), the present model predicts probabilistically the spatial characteristics of a dynamic microtubule array without simulating the dynamics of individual microtubules. That a probabilistic description of a *dynamic* array is predicted needs emphasizing. A more explicitly kinetic approach to establishing a measure of efficiency of the dynamic microtubule array in covering the intracellular space is to calculate the distributions of first-passage times [35]. In the present context of the microtubule-organelle contact, these could be called first-contact times. An alternative formalism, which would be a natural extension of the referenced line of work that used the Monte Carlo technique [18], [19], [22], would rely on a discrete representation of the microtubule cytoskeleton. In a model of this type, a given simulated microtubule would either be present (probability 1) or not present (probability 0) at a given point in space, and it would alternate between these states in the course of the simulation. Using this approach, the microtubule dynamics would need to be explicitly simulated over a long time to obtain the non-zero time-averaged densities. The formalism used here calculates these densities directly and analytically. The theory developed here operates with probabilistic number densities, such as those of microtubule ends (Equation 4) and of microtubules (Equation 10). These are continuous functions that take non-zero values everywhere within the modeled domain of intracellular space. The positive probability density at any point in space reflects quantitatively the likelihood that microtubules in the real (discrete and dynamic) structure extend to or through this point. Thus, although it is not the only possible modeling approach to the problem at hand, the modeling technique chosen here is adequately efficient at predicting the spatial properties of a dynamic microtubule array.

## Methods

The described calculations were performed using Mathcad software (Parametric Technology Corporation, Needham, MA).
